# A genome‐wide epitranscriptomic regulatory atlas in the human aging brains with Alzheimer's disease

**DOI:** 10.1002/alz70855_107305

**Published:** 2025-12-24

**Authors:** Amit Kumar Gupta, Andrew A. Pieper, Andrew J. Saykin, Feixiong Cheng

**Affiliations:** ^1^ Cleveland Clinic, Cleveland, OH, USA; ^2^ Cleveland Clinic Genome Center, Cleveland, OH, USA; ^3^ Case Western Reserve University, Cleveland, OH, USA; ^4^ Institute of Transformative Molecular Medicine, Case western Reserve University, Cleveland, OH, USA; ^5^ Louis Stokes Cleveland VA Medical Center, Cleveland, OH, USA; ^6^ Brain Health Medicines Center, Harrington Discovery Institute, University Hospitals Cleveland Medical Center, Cleveland, OH, USA; ^7^ Indiana Alzheimer's Disease Research Center, Indiana University School of Medicine, Indianapolis, IN, USA; ^8^ Department of Radiology and Imaging Sciences, Center for Neuroimaging, School of Medicine, Indiana University School of Medicine, Indianapolis, IN, USA; ^9^ Department of Medical and Molecular Genetics, Indiana University School of Medicine, Indianapolis, IN, USA; ^10^ Stark Neurosciences Research Institute, Indiana University School of Medicine, Indianapolis, IN, USA

## Abstract

**Background:**

RNA editing is at the forefront of epitranscriptomic modification, associated with several functions from regulating RNA functions and alternative splicing to its role in neuronal dynamics and immune response. Studying RNA editing signals could pave the way for identifying potential biomarkers and therapeutic modalities in Alzheimer's disease (AD).

**Method:**

We performed comprehensive analysis of RNA editing events in multiple human aging brain tissues linked with AD adjusted for age, postmortem interval, sex, and *APOE4* status implementing REDItools and rnaEditr package. We have utilized transcriptomic, whole genome sequencing (WGS), and genome wide association study (GWAS) data to determine key RNA editing loci, heterogeneities, and regulatory implications in AD. Briefly, genotype data was first quality filtered, assembly converted, and annotated utilizing PLINK, CrossMap and bcftools, respectively. Further, RNA editing QTLs (edQTLs) were identified using MatrixEQTL and GTEx_edQTL packages. Later, colocalization analysis has been performed employing coloc (Bayesian function) using edQTLs and AD GWAS summary statistics.

**Result:**

Brain‐wide RNA‐editing events and edQTLs substantiated with GWAS data highlights 51 significant RNA editing loci pertaining to 26 genes, conserved across multiple brain regions in AD. Key examples of RNA editing events found in well‐known AD genes include *CLU* (rs7982‐chr8:27604964, FDR=1.07×10^‐48^, PP4≥0.70) and *BIN1* (rs2276582‐chr2:127054551, FDR=1.28×10^‐36^, PP4≥0.99), linked with β‐amyloid formation, lipid transport, tauopathy, and neurotransmission. Another key editing signal (rs10417824‐chr19:1009884, FDR=7.46×10^‐40^, PP4≥0.99) found significant across all nine brain regions in *GRIN3B* (glutamate ionotropic receptor NMDA type subunit 3B), a critical excitatory neurotransmitter in the brain that also undergoes extensive alternative splicing, generating multiple isoforms which could be due to RNA editing events. Correspondingly, neuronal tyrosine phosphorylated phosphoinositide‐3‐kinase adaptor 1 (*NYAP1*) crucial for neuronal migration and morphogenesis has significantly regulated editing site (rs12539172‐chr7:100494172, FDR=6.68×10^‐97^, PP4≥0.99). Similarly, an RNA editing locus (rs2734897‐chr7:100577850, FDR=8.43×10^‐110^, PP4≥0.80) in *AGFG2* (ArfGAP with FG repeats 2) indicating a role in amyloid metabolism. Overall, brain‐wide epitranscriptomic functional and regulatory results allude to amyloid/tau regulations, neuroinflammation, neurotransmission, protein metabolism, and immune system function in AD.

**Conclusion:**

These emerging findings present likely implications for RNA editing in AD pathogenesis, providing potential biomarkers and therapeutic targets for AD and other aging‐related dementia if broadly applied.

